# An update of “Cost-effectiveness of rotavirus vaccination in the Netherlands: the results of a Consensus Rotavirus Vaccine model”

**DOI:** 10.1186/1471-2334-13-54

**Published:** 2013-01-30

**Authors:** Hong Anh T Tu, Mark H Rozenbaum, Pieter T de Boer, Albert C Noort, Maarten J Postma

**Affiliations:** 1 Institute of Health Policy, Management and Evaluation, University of Toronto, Ontario, Canada

**Keywords:** Cost-effectiveness analysis, Rotavirus vaccination, the Netherlands

## Abstract

**Background:**

To update a cost-effectiveness analysis of rotavirus vaccination in the Netherlands previously published in 2011.

**Methods:**

The rotavirus burden of disease and the indirect protection of older children and young adults (herd protection) were updated.

**Results:**

When updated data was used, routine infant rotavirus vaccination in the Netherlands would potentially become an even more cost-effective strategy than previously estimated with the incremental cost per QALY at only €3,000-4,000. Break-even total vaccination costs were indicated at €92–122, depending on the applied threshold.

**Conclusions:**

We concluded that the results on potentially favourable cost-effectiveness in the previous study remained valid, however, the new data suggested that previous results might represent an underestimation of the economic attractiveness of rotavirus vaccination.

## Background

In 2011, we published the study “Cost-effectiveness of rotavirus infection in the Netherlands: the results of a consensus model” in BMC Public Health [1]. The study focused on the Dutch situation using the Consensus Rotavirus Vaccine (CoRoVa) model developed by the University of Groningen, the Netherlands. The CoRoVa model is a cohort simulation model capturing the epidemiology of rotavirus infection and economics of vaccination. Since its publication, several studies focusing on the disease's epidemiology and on the observed indirect protection (herd immunity) after the introduction of the vaccine have been carried out in other countries. In particular, there have been studies on the etiology of acute gastroenteritis in hospitalized children in the Netherlands [2,3]. In these studies, the incidence and relevance of rotavirus reported a national number exceeding 5,000 hospitalized rotavirus gastroenteritis (RVGE) cases among children less than 5 years of age. These newly reported national totals of RVGE cases are much higher than the ones reported previously [1,4-7]. Notably, in our previously published study, we used the epidemiological data of 3,600 hospitalized RVGE cases, which was conservatively retrieved from Mangen et al. [8] on the basis of a re-analysis of raw data from previous epidemiological studies [4,7,9]. The inclusion of the new hospitalization data of RVGE cases for our re-analysis would obviously make rotavirus vaccination more favorable than previously calculated in several studies [1,6,7,10,11]. In addition to epidemiological studies of rotavirus-related disease, studies on the observed indirect protection (herd immunity) have been carried out either through observing the actual change in RVGE hospitalized cases in the US [8,12] between pre and post- rotavirus vaccination eras or through projecting the impacts of rotavirus vaccination by applying mathematical transmission models in England and Wales [13] or in five other countries in the European Union [14]. The results from these studies have reinforced results of other studies on the topic [8,15-20]. It is important to emphasize that the US-study on herd protection reported the observation of an actual decrease in the annual RVGE hospitalized cases for all age groups after rotavirus vaccination was introduced in the US in 2006 [8,12,21]. This was the major difference compared to studies from other countries where indirect protection was predicted based on mathematical modeling of vaccination of a hypothetical cohort.

Factoring all of these, we decided to use both the new hospital data and the evidence on herd immunity in our updated analysis of the CoRoVa model. With our analysis we hope to contribute to the ongoing discussion on the decision of implementing routine rotavirus vaccination in the Netherlands taking into account estimation of the incremental cost-effectiveness ratio (ICER) play an important role in this discussion.

## Methods

We adjusted our model to reflect the new and higher incidence of rotavirus gastroenteritis and included the indirect protection for individuals aged 5 years and older using data from Friesema et al. [7] and Lopman et al. [12]. In the previously published study, we also included herd immunity but only in one specific scenario analysis and only for individuals aged less than 5 years. In the current analysis, herd effects for children under five years of age were calculated exactly as it was done in the original study. It meant we assumed herd protection benefits for those not yet (fully) protected by the vaccine (either too young to be vaccinated or those who had not yet received the complete set of doses) and non-vaccinated children (5% of a birth cohort for the Dutch situation), assuming protection would be as effective as the vaccination would be after the completion of all doses [1]. To account for herd effects for individuals of 5 years and older, we used data from Lopman et al. [12], where the rotavirus discharge rates for different age groups, e.g., 5–14; 15–24; 25–65 and ≥ 65 years old were compared between the post and prior rotavirus vaccination periods. However, we decided to include herd protection only for age groups of 5–14 and 15–24, where there were statistically significant changes in reduced RVGE hospitalized cases [12]. In order to capture this indirect protection resulting from rotavirus vaccination, we have reduced the incidence of hospitalized cases due to rotavirus infection by the same rate as indicated by the US rotavirus discharges. In details, the authors reported the change of RGVE hospitalized cases comparing the pre- (2000–2006) and post-vaccination eras (2008) using the relative risk (RR). The RRs for rotavirus-coded hospitalization discharges in 2008 compared with the prevaccine period of 2000–2006 were 0.29, 0.35 for age groups of 5–14 and 15–24, respectively. The choice to base the indirect effects on this specific study was made as it reported the actual decrease in RVGE cases from a large database covering ~20% of all US hospital admissions after 2-year follow up since the introduction of rotavirus vaccination in the US in 2006. We felt that using these actual epidemiological data was a conservative approach and would provide a better picture of the impact of rotavirus vaccination as it also reflected the change in vaccine uptake. With the increasing evidence of herd protection in unvaccinated or otherwise unprotected children below 5 years of age, we also focused on the CoRoVa-model scenario that included that type of herd protection [8,12,17-19].

In the original study, we included conservative incidence numbers based on the data provided by Mangen et al. [6], where the RVGE hospitalized cases were estimated at 3,600 [6,7]. As mentioned, recent studies indicated relevantly higher annual hospitalizations of at least 5,000 cases below 5 years of age. Conservatively, this lower bound was used in the analyses as ballpark figure for total rotavirus hospital cases (including nosocomial infections) in the Netherlands [3]. For the sake of comparability, we recalculated results applied to the birth cohort of 180,000 newborns as used in the original publication. In order to calculate the age-specific disease distribution, we applied the age-specific hospitalization distribution which divided the total estimated number of cases by the different age groups exactly as indicated in the original study [1]. The relative increase for the hospitalizations <5 years was also applied to those above 5 years of age (which may again be considered conservative [12]).

Except for updating the new hospitalized RVGE cases and including the herd effects, we applied the same vaccine efficacy, waning vaccine protection, Dutch discount rates for health effects and costs of 1.5% and 4%, respectively; 2010 consumer price index acquired from the Netherlands’ Central Bureau of Statistics, no care-giver quality of life loss and treatment cost for rotavirus cases of all categories, as we did in the original study [1]. In the absence of the vaccine price, we applied a total cost of €75 per fully vaccinated child. The threshold willingness-to-pay was conservatively assumed to be €20,000 per quality-adjusted-life-year (QALY), with additional calculations at €50,000/QALY [19]. All the analyses were done from the societal perspective, inclusive parental work absenteeism, however excluding indirect costs from any deaths in infants due to rotavirus according to the Dutch friction costing method for indirect productivity costs [1]. As there was no data on QALYs for children and adults five years and older and there were no deaths reported due to rotavirus infection for older children and adults, we only estimated the direct and indirect costs saved due to hospitalized cases and conservatively excluded any QALY effects.

## Results and discussion

With the inclusion of the new hospital incidence data, vaccination could prevent 4,008 cases of community acquired hospitalizations and 599 cases of nosocomial cases (Table 1) for children under five years of age. This lead to a decrease of 1,544 community acquired hospitalizations and 284 nosocomial hospitalizations compared to the original study (Table 1). These additional prevented cases translated to an additional gain of 61 QALYs and an extra saving of appropriate €4.5 million on direct medical and indirect non-medical costs of parental productivity losses. Extra gains in QALYs and additional savings would reduce the ICER to €3,800 compared to €46,700 in the original study (Table 2). Importantly, in the original study, when herd protection was considered for children under five years of age, the ICER was already €28,400 [1]. When herd immunity was considered in all age groups below 25 years, rotavirus vaccination corresponded to an ICER of €3,200 despite the exclusion of utility estimates for individuals aged 5 years and older experiencing rotavirus infection, any possible deaths in these older individuals and potential indirect costs.

**Table 1:** Results on hospitalized RVGE cases, costs and QALYs for the original study and with new hospitalization data and herd protection

	**No vaccination**		**Vaccination**		**Difference**	
	**Individuals aged <5y**	**Individuals aged ≥5y**	**Individuals aged <5y**	**Individuals aged ≥5y**	**Individuals aged <5y**	**Individuals aged ≥5y**
**Number of hospitalized cases from the original study**^**1**^						
Community accquired	2,817	161	353	161	2,464	0
Nosocomial	421	149	106	149	315	0
**Number of hospitalized cases from the original study (including herd protection for children <5 years)**						
Community accquired	2,817	161	155	161	2,662	0
Nosocomial	421	149	23	149	398	0
**Number of hospitalized cases using the updated hospitalization data (and herd protection for <5 years)**^**2**^						
Community accquired	4,241	223	233	223	4,008	0
Nosocomial	634	207	35	207	599	0
**Number of hospitalized cases using the updated hospitalization data and herd protection (for <25 years)**^**2**^						
Community accquired	4,241	223	233	180	4,008	43
Nosocomial	634	207	35	167	599	40
**Discounted cost and QALYs from the original study**^**1**^						
Total costs (direct and indirect)^3^	9,663,000	6,519,000	2,074,000	6,519,000	7,589,000	0
Total QALYs	173	NA^4^	64	NA^4^	109	NA^4^
**Discounted cost and QALYs from the original study (including herd protection for <5 years only)**						
Total costs (direct and indirect)^3^	€9,663,000	€6,519,000	€969,000	€6,519,000	€8,694,000	€0
Total QALYs	173	NA^4^	32	NA^4^	141	NA^4^
**Discounted cost and QALYs using the updated hospitalization data (and herd protection <5 years)**^**2**^						
Total costs (direct and indirect)^3^	€13,246,000	€6,937,000	€1,203,000	€6,937,000	€12,043,000	€0
Total QALYs	207	NA^4^	37	NA^4^	170	NA^4^
**Discounted costs and QALYs using updated hospitalization data and herd protection (for <25)**^**2**^						
Total costs (direct and indirect costs)^3^	€13,246,000	€6,937,000	€1,203,000	€6,833,000	€12,043,000	€104,000
Total QALYs	207	NA^4^	37	NA^4^	170	NA^4^

^1^Herd protection was not included in the base case analysis; ^2^Herd protection for individuals aged less than 5 years of age was included in the same way as in the scenario analysis of Rozenbaum et al. [1]; ^3^Costs are excluding vaccination costs; ^4^No utility losses were available for individuals aged 5 years and older and death was conservatively assumed to occur in individuals aged less than 5 years of age only in the absence of any reported deaths in older ages.

**Table 2:** Results on cost-effectiveness for various scenarios

**Scenario**	**ICER in €/QALY**^**1**^	**Threshold at €20,000 per QALY:vaccination costs per child**	**Threshold at €50,000 per QALY:vaccination costs per child**
**Results from the original study (no herd immunity)**	46,700	**€**58	**€**77
**Results including updated hospitalizations (no herd protection)**	15,600	**€**78	**€102**
**Results including updated hospitalizations with herd protection in <5 years**	3,800	**€**91	**€122**
**Results with updated hospitalizations and herd protection in <25 years**	3,200	**€92**	**€122**

^1^Total vaccination costs of €75 per fully vaccinated child assumed.

## Conclusion

We updated our initial analysis of the cost-effectiveness of rotavirus vaccination in the Netherlands [1]. We concluded that at the assumed total vaccination cost of €75 per child the new hospitalization data and herd immunity included, rotavirus vaccination would be much more cost-effective compared to the results of the original study. The incremental cost was only between €3,000-4,000 per QALY. Highly favorable cost-effectiveness already results for total vaccination costs below *€*92. We acknowledged that our analysis was based on a static decision model, which was limited in comparison to dynamic transmission models, with the latter enabling dynamic estimation of indirect protection from vaccination. Yet, it might be argued that the static approach generally provides an underestimate of the attractiveness of vaccination in economic terms. Given our overall conservative approach, our results are likely to underestimate the true cost-effectiveness. Yet, we feel that further work directed to synthesizing all the above information into a to be developed dynamic model is important for further work to be undertaken. Our new piece of information is crucial for the ongoing discussion in the Netherlands concerning the possible inclusion of rotavirus vaccination in the Dutch national immunization program and at what price to be tendered.

## Abbreviations

CoRoVa-model: Consensus Rotavirus Vaccine model; ICER: Incremental cost-effectiveness ratio; RVGE: Rotavirus gastroenteritis; QALY: Quality-adjusted life year.

## Competing interests

Prof Postma received grants, honoraria and travel stipends from various pharmaceutical companies, inclusive those manufacturing the rotavirus vaccines. This study was supported by an unrestricted grant form SPMSD (Hoofddorp, Netherlands). M Rozenbaum is an employee of Pfizer (Capelle, Netherlands) since fall 2011, next to his guest researchship at the University of Groningen. The other authors have no competing interests to report.

## Authors’ contributions

HATT and MHR analyzed the data, interpreted the results and reported the findings. MJP supervised the project and drafted the manuscript together with HATT and MHR. PTB and ACN critically reviewed the data and draft manuscripts. All authors read and approved the final manuscript.

## Pre-publication history

The pre-publication history for this paper can be accessed here:

http://www.biomedcentral.com/1471-2334/13/54/prepub
